# Improving cervical cancer continuum of care towards elimination in Ethiopia: a scoping review

**DOI:** 10.21203/rs.3.rs-2791526/v1

**Published:** 2023-04-14

**Authors:** Aklilu Endalamaw, Habtamu Alganeh, Muluken Azage, Asmamaw Atnafu, Daniel Erku, Eskinder Wolka, Adane Nigusie, Anteneh Zewdie, Destaw Fetene Teshome, Yibeltal Assefa

**Affiliations:** Bahir Dar University; Bahir Dar University; Bahir Dar University; University of Gondar; Griffith University; International Institute of Primary Health Care Addis Ababa; University of Gondar; International Institute of Primary Health Care Addis Ababa; University of Gondar; University of Queensland

**Keywords:** Attitude, Cervical Cancer, Knowledge, Mortality, Perception, Ethiopia

## Abstract

**Introduction:**

Cervical cancer is the second-leading cause of death among all cancers in Ethiopia. Ethiopia plans to eliminate cervical cancer as a public health problem by 2030, following the World Health Organization’s call for action. A scoping review was conducted on the status of the cervical cancer continuum towards elimination in Ethiopia.

**Methods:**

We searched articles in PubMed, Scopus and Google Scholar. All studies conducted on cervical cancer in Ethiopia, irrespective of date of publication, type of article, or language of publication were included. However, conference abstracts, commentaries, and letters to the editors were excluded. We used EndNote x9 software to merge articles from different databases and automatically remove duplicates. Screening of titles, abstracts, and full texts was performed by two co-authors independently. The cancer care continuum was employed as a framework to guide data synthesis and present the findings.

**Results:**

Of the 569 retrieved articles, 159 were included in the review. The found most of articles were about knowledge, attitude, and practice. There were few studies on health-seeking behaviour, perception and acceptability to cervical cancer services and availability and readiness of a screening programme. The review identified that there was inadequate knowledge, attitude and perception about cervical cancer. Screening for cervical cancer is not widely used in Ethiopia. Knowledge and attitude, education status, and income were repeatedly reported as precursors for cervical cancer screening. Most studies concluded a high prevalence of precancerous lesions and cervical cancer, as well as high mortality rates or short survival times. The review also identified that there is huge heterogeneity in findings under each component of the cancer care continuum across time and geographic settings.

**Conclusions:**

Overall, there is inadequate knowledge, perception, health seeking behaviour, screening and treatment services. This implies that the country is lagging behind the targets towards eliminating cervical cancer despite the availability of effective interventions and tools. We argue that an implementation research is needed to identify implementation issues, challenges and strategies to scale up both primary and secondary prevention services so that cervical cancer will not anymore be a public health problem.

## Introduction

Cervical cancer is a significant global health problem, with an estimated 604,127 new cases and 341,831 deaths worldwide in 2020, making it the fourth-most prevalent cancer among women (1). Almost 90% of these deaths occur in low- and middle-income countries (2). Africa has seen the highest incidence and mortality rate of cervical cancer, and countries in the region have bear a greater burden compared to others (3). For instance, in Ethiopia, there were 7,445 new cases of cervical cancer and 5,338 deaths in 2020 (4). Cervical cancer has a huge impact women’s disability-adjusted life years, financial burden, and life expectancy (5, 6). Cognizant of its impact, global and national policy strategies have been set as a guide to halt the incidence and deteriorating effects of cervical cancer (7).

There is ample evidence that vaccination, early identification, and treatment are effective to prevent cervical cancer (7). In line with this, the World Health Organization (WHO) launched a plan to eliminate cervical cancer as a public health problem by 2030 through targets including 90% of girls fully vaccinated with human papilloma virus (HPV vaccine) by age 15 years, 70% of women are screened with a high-performance test by 35 years of age and again by 45 years of age, and 90% of women identified with cervical disease receive treatment (90% of women each precancer treated and invasive cancer managed) (8). The Economist Intelligence Unit has proposed recommendations to eliminate cervical cancer as a public health treat, including generating local data to inform health financing design and decision-making (9). The Union for International Cancer Control also supports prevention, multi-cancer early detection, and treatment in support of the WHO cervical cancer elimination policy (10).

Despite these global initiatives and recommendation, the progress towards cervical cancer elimination is still lagging behind those targets. A study in Ethiopia found that women had a relatively poor awareness of cervical cancer and favoured traditional remedies as a treatment option for the early stages of the disease (11). Inadequate awareness is one of the challenges to receiving health care services (12).

The strategic plan for cervical cancer elimination calls for countries to adopt a full-continuum approach, encompassing prevention, screening, diagnosis, treatment, psychosocial care, and outcomes (13). However, there is scarcity of evidence about these cancer continuum indicators, making it difficult to determine the status of program implementation towards elimination. We argue that an in-depth understanding of the overall cervical cancer continuum, including epidemiology, promotion, prevention services, as well as outcomes and impacts will help countries to develop mid- and long-term strategy towards elimination.

In Ethiopian context, this scoping review was conducted to assess the cervical cancer continuum in Ethiopia, including prevalence of cervical cancer, knowledge, attitude, perception, health-seeking behavior, prevention services, and outcome and impacts of cervical cancer.

## Methods And Materials

### Reporting

The review was conducted based cancer care/control continuum model (13). The first component is health promotion and disease prevention services/domains. These are: behavioural interventions (awareness, knowledge, attitude, and perception), vaccination, and the health-seeking behaviour of women. Screening and diagnosis are the second phase in the continuum. Psychosocial care encompasses the cancer patient’s experience with treatment, satisfaction, and general happenstance during their interaction with the health system. Furthermore, treatment outcome and disease impact are important section of the continuum (Figure).

### Search strategy

A literature search was conducted using the term cervical cancer associated with Ethiopia, irrespective of language, settings, and date of publication. We searched articles indexed in PubMed and Scopus from inception to March 15, 2023. We searched Ethiopia with ‘AND’ Boolean operator in the title and abstract of records in PubMed, and in the title, abstract, and keywords of records in Scopus. Additional records were searched on Google Scholar.

### Inclusion and Exclusion criteria

Inclusion criteria were any kind of studies conducted on cervical cancer in Ethiopia. There was no restriction on the date of publication, type of article, or language of publication. However, conference abstracts, commentaries, and letters to the editors were excluded.

### Screening, selection and data extraction

We used EndNote x9 software to merge search results from different databases and remove duplicates. Screening of articles by titles, abstract and full text was conducted independently by two of the co-authors (AE and HA). Any disagreement was solved by discussion. A data extraction sheet was prepared and approved in consultation with the last author (YA). The data extraction sheet included first author, publication year, study setting, region, study design, major category (based on the cancer care continuum), and main findings.

### Data synthesis

The characteristics of the articles were described using frequency distribution. Data synthesis began with a thematic category of available articles based on the cancer care continuum. These themes were knowledge and attitude or perception towards cervical cancer or prevention services, HPV, health seeking behaviour, diagnosis (prevalence of cervical cancer lesions), treatment (health care experience), and outcome (mortality).

## Results

### Characteristics of articles

The search identified a total of 569 articles: 248 from Scopus, 158 from PubMed, and 164 from Google Scholar. After title, abstract and full-text screening, 159 articles were included (supplementary file on characteristics of articles) in the review. Eleven articles were on HPV vaccination, with different proportions in the regions. There were 36 articles on knowledge, attitude, and practice about cervical cancer screening. The willingness and acceptability of women for cervical cancer screening were examined by one study each in Amhara and Addis Ababa. Four articles assessed health-seeking behaviour and demand for cervical cancer screening (2 articles in Addis Ababa and one article each in Oromia and Tigray). Five articles assessed women’s intention to undergo cervical cancer screening in Amhara (3 articles) and Oromia (2 articles). Forty-two articles were available on cervical cancer screening uptake. In 26 articles, the epidemiology of precancerous cervical lesions is described, and the prevalence of cervical cancer is assessed in 6 articles. Ten articles were found on cervical cancer patients experiences when interacting with the health system or due to the disease mechanism. Ten articles (seven studies in Addis Ababa and three in the Amhara region) were found on mortality or survival of patients with cervical cancer. This review findings presented based on the cancer care continuum model in the subsequent sections.

### Health Promotion and Prevention

#### Knowledge, attitude/perception and practice

The findings related to knowledge, attitude, perception and practice are heterogeneous among populations and locations. Among university/college students good knowledge about cervical cancer/and/or screening ranged from 9.3% (14) to 35.6% (15). Knowledge about the cause and risk factors of cervical cancer was also low among health care providers; only 43.8% of female healthcare providers were knowledgeable (16), while 51.6% of urban health extension workers were non-knowledgeable (17). Among women who attended health facilities, 21.2% in the Amhara region (18) and 86.9% in the Southern Nations Nationalities Region (SNNPR) (19) were knowledgeable. According to community-based studies, around 20% of women in Amhara region (20) and 46.4% of women in Tigray region (21) had knowledge about cervical cancer.

The review findings were also observed in attitude towards cervical cancer and/or screening among college or university students, health care professionals, women in the community, and those who attended health care facilities. It ranged from 44.1% at Wollega University (22) to 71.7% at Adama Science and Technology University (23). About 30.7% of female health care providers had unfavourable attitude in the Amhara region (16). Based on community-based studies, the lowest result (53.3%) and highest (64%) of women had a favourable attitude towards cervical cancer in Tigray (21) and Amhara regions (24), respectively. A lower level (46.1%) of favourable attitude was reported among women who attended health facilities (25). More than one-third (34.8%) of women in the SNNPR had unfavourable attitude towards cervical cancer (26).

Few studies assessed the perception of university students, women, and men, as well as community leaders on cervical cancer and/or screening. For example, 33.2% of female students in higher education perceived to be at risk of cervical cancer (15). According to a qualitative study, women in the community perceived that they were not vulnerable to the disease and believed cervical cancer screening was not necessary (27). Socioeconomic and demographic barriers are available in supplementary table (sTable 1).

#### Health seeking behaviour

A qualitative study among men, women, and community leaders in rural settings in Oromia region revealed that study participants preferred traditional healers over modern medicine (28). Women’s health seeking behaviour was also found to be low in Addis Ababa (29). It was found that only 14.2% of women had good health seeking behaviour in the southern part of Ethiopia (30). Lack of information and awareness, or poor knowledge, were the common barriers or reasons that women and community leaders were unable to attend health care (28–30) (sTable 2).

#### Human Papilloma Virus Vaccination

The review found that that HPV vaccines acceptance rate of parents varied from 44.8% (31) to 94.3% (32). The HPV vaccination status among female students ranged from 44.4% (33) to 66.5% (34). Higher acceptance of vaccine was associated with media exposure and parents’ perceptions of positive behavioural control (35). On the other hand, lack of knowledge (11, 36), unfavourable attitudes toward the vaccine (37, 38), living in rural area, poor socioeconomic status, and being a member of an unidentified target population for HPV vaccination (31) were identified as challenges of the vaccination programme (sTable 3).

### Early detection and diagnosis

#### Cervical cancer screening

A meta-analysis, based on the articles published before 2020, estimated that 14.8% of women utilized cervical cancer screening services in Ethiopia, with the lowest in the Amhara region (13.6%) and the highest in the SNNPR (18.6%) (39). Another meta-analysis showed 18.2% of women living with human immunodeficiency virus (HIV) underwent cervical cancer screening in 2020 (40). There were studies that reported that 2.5% (14) or none (22) of university students were screened for cervical cancer. About 20.3% of female sex workers responded that they had received cervical cancer screening (41).

One nationwide study among 632 health facilities using service availability and readiness assessment 2016 and 2018 involving ‘equipment’, ‘reagent’, ‘training’ and ‘guideline’ as tracer items. This study found that 21% and 33% of the health facilities delivered cervical cancer screening in 2016 and 2018, respectively; none of health facilities have fulfilled all four components (42). Moreover, health care providers have rare exposure to demonstrate cervical cancer screening skills. For example, only 8.7% of female health care providers had exposure to regular cervical cancer screening practises in Amhara region (16).

The review also identified effective strategies to scale up the cervical cancer screening programme. These were a cervical cancer screening campaign assisted by robust technology (43), health education at the coffee ceremony (44) and other settings (45), and ‘single-visit approach’/ ‘Addis Tesfa Project’ (46). Individual and social determinants that encouraged utilization of cervical cancer screening were having symptoms of vaginal bleeding, physician recommendation (47), women having attended formal education and having good knowledge toward cervical cancer screening, a history of sexually transmitted infections (39), a favourable attitude about cervical cancer and screening (48) and higher sexual autonomy (49). Another study involving health workers also identified barriers such as low community and provider awareness of cervical cancer, lack of space and equipment to offer screening, and lack of support from leaders (50), social and religious influence (51). The detail characteristics of the studies and additional findings are available in sTable 4.

#### Precancerous cervical lesion and cervical cancer

The overall prevalence of oncogenic HPVs (HPV 16/18) and the VIA-positivity rate, possibly indicative of cervical lesions, were 7.1% and 13.1%, respectively (52). Another study using VIA estimated that 10.3 % (53) and 23.1% (54) of women who attended hospitals in Addis Ababa had indicative of cervical cancer lesion. The second study reported cervical cancer lesion among women who attended Gahandi Memorial Hospital between 2015 and 2019 (54). Precancerous lesions were reported in different settings of the Amhara region, ranging from 5.3% (55) to 9.9% (56). Cervical cancer prevalence was 15.6 per 1000 in the general population (all women) in 1992 (57) and 7.5% of women living with HIV who attended Addis Ababa Specialised hospitals had a precancerous lesion in 2022 (58). The former study revealed that 33 out of 2111 women underwent a clinical and cytological investigation in hospitals and clinics in Addis Ababa (57) (sTable 5).

### Treatment and Psychosocial care

#### Cervical cancer patients’ or care givers’ experience

Cervical cancer patients have experienced multiple challenges when they pass through the screening and treatment phases. Women waited a long time to receive a pathology-based cervical cancer diagnosis; the median time was 30 weeks (59) and 137 days for radiotherapy care (60). Women also experienced late diagnosis examination for cervical cancer in Addis Ababa; 86.3% of women had delayed diagnosis of cervical cancer (61).

Only 41% of women who underwent cervical cancer screening in Addis Ababa were satisfied with the services (62). Women with cervical cancer faced interruptions of social cohesion, felt stressed, and struggled with work and daily life (63); Care givers were also less satisfied with ‘physical patient care’ and ‘provision of information’ (64). Cervical cancer results in social impact on patients, including social discrimination (61.8%), loss of body image (63%), loss of sexual functioning (78%), loss of femininity (89%) and financial crises by loss of income (45.7%), medical and non-medical expenditure (71%), and work and employability challenges (66.8%) (65). The quality of life of women diagnosed with cervical cancer was low due to poor physical functioning, emotional functioning, pain, and symptom experience (66). Two studies showed the financial costs of cervical cancer (63, 67). One of the two articles that reported financial crises qualitatively explored women’s experiences during their follow-up care in Tikur Anbessa Specialized Hospital, Addis Ababa in 2022 (63). The latter study estimated the direct outpatient and inpatient costs that women with cervical cancer incur for health care was US$334.2 and US$329, respectively, in 2013 (67).

### Outcome

#### Mortality due to cervical cancer

There are reviews on mortality due to cervical cancer that assessed using document reviews in hospitals. In Addis Ababa, only 28% of study participants survived in five years (68). Another study estimated that 38.6% of women survived for five years (69). The incidence of mortality was 15.6 per 100 per year (70). In contrast, the overall incidence of mortality was estimated to be 31 per 100 person-years of follow-up (71). In the Amhara region, 36.6% of women died in a study conducted at the University of Gondar Hospital from 15 May 2018 to 15 May 2022; they also estimated that the median survival time was 42 months (72). Another study in the same setting estimated that probability of women to death was increased with an increased tumor size (73). Another study in Felegehiwot Comprehensive Specialised Hospital in the Amhara region estimated that the mean survival time was 40.1 months from 25 June 2017 to 31 March 2021 (74). Adherent patients were more likely to survive (75), while older age groups and women with advanced disease stages and low baseline anaemia were more likely to die (76) (sTable 6).

## Discussion

This scoping review, conducted based on the cancer continuum care model on women’s knowledge, attitude, and perception, health seeking behaviour, uptake of cervical cancer prevention services (HPV and screening), prevalence of cervical cancer, women’s experience in health care, and treatment outcome (e.g., mortality) in Ethiopia.

The majority of the articles reported that about 50% and more of the study participants were not knowledge about cervical cancer, prevention, and services (19, 22, 77, 78). Similarly, about half or more women had an unfavourable attitude except in a few studies that reported better attitude coverage (14, 23, 77, 79, 80). Knowledge was frequently reported as a precursor to a favourable attitude (16, 26, 81). A meta-analysis finding among women living with HIV in Africa revealed a comparable estimate to knowledge (43.0%) and attitude (38.0%) towards cervical cancer (82). Lack of information and awareness, or poor knowledge, was also the common barrier or reason that women and community leaders were unable to attend health care (28–30) and the reason for unacceptability of screening services (83, 84). In contrast, a meta-analysis finding in Africa claimed that ‘school-based education’ improves knowledge, perception, attitude, intentions toward and uptake of cervical cancer screening services (85). The review also found that women’s and communities’ health-seeking behavior was low, including among those living in the capital city, Addis Ababa. Cervical cancer screening acceptability was low among women with HIV in Addis Ababa (83) and a rural small city in Amhara region (84) that were conducted before nine and six years, respectively, which needs recent research. Additionally, further triangulated evidence, including men, women, and key informants from several sectors, on knowledge, attitude, and perception would support the implementation of whole-of-society and whole-of-government approach to strengthen and scale up cervical cancer prevention activities. at all levels (86).

A higher HPV vaccination coverage was observed among students; both were above the global estimation; global HPV vaccination coverage was 12.2% in 2018 (87). Another meta-analysis finding in low-and middle-income countries (45.48%) (88) is comparable with vaccination uptake based on one study in Oromia region in Ethiopia (44%) (33), but lower than a single district study in Amhara region Ethiopia (66.5%) (34). However, HPV vaccination coverage was high in routine or administration programmes (89.0%) (88). Those female adolescents who had prior information, good knowledge, and a favourable attitude were more likely to be vaccinated and show a willingness to be vaccinated (33, 34). HPV vaccination coverage was further improved by narrative education, outreach plus reminders, financial incentives plus reminders, brief motivational behavioral interventions, training plus assessment and feedback, and multicomponent interventions in high-income countries (89). These are relevant interventions that could assist the scale up of vaccination program in Ethiopia. However, it should be guided by implementation studies that evaluate the effectiveness, efficiency, and acceptability of these interventions in Ethiopia. Though few studies found a higher percentage of women showing willingness to get their children vaccinate in Ethiopia (32), many women did not participate in health care interventions for their own health, as a higher percentage of women were not screened for cervical cancer (90).

The review found that the national coverage of cervical cancer screening was low (39, 48, 91). Cervical cancer screening was slightly higher among women with HIV (40) than other women (39, 91); this might be because women with HIV have better exposure to health services due to long-term adherence with HIV treatment. A worldwide review showed that about 67% of women aged 30–49 years have never been screened for cervical cancer, which is very low in low-income countries (11%) (92). Women’s income was associated with cervical cancer screening (93) as were knowledge, attitude, and health-seeking behavior (17, 19, 21, 94). The cost-effectiveness of cervical cancer screening methods (cytology-based screening and provider-collected HPV testing, or VIA) was assessed in low- and middle-income countries (95), which identified that HPV test was cost efficient and effective that cytology-based screening. Similar evidence is needed in Ethiopia to identify effective and cost-effective interventions for cervical cancer screening and treatment. It is worrisome that this low and unsatisfactory cervical cancer screening happens despite the high rate of precancerous cervical lesions and cervical cancer in Ethiopia. There have been well-known effective strategies, including technology assisted screening campaign (43), health education at the coffee ceremony (44) and a health education intervention (45), and ‘single-visit approach’ (46). Implementation of these strategies could be challenged by low-community and provider awareness, lack of infrastructure, lack of screening machine, and high staff turnover in Ethiopia (50). In another lower-income country, Bangladish, there are similar supply-side barriers, for instance, lack of skilled screening providers, lack of advocacy and health promotion, resource constraints, lack of effective leadership (96). These strategies to be implemented and barriers to be solved should be evaluated further for their acceptability and cost-effectiveness.

The review also found that the prevalence of cervical cancer was high in Ethiopia (54, 97) compared to many low-income countries (98). Cervical cancer, including precancerous lesions, is high in a country where a higher percentage of women practised multiple sexual intercourse and had a history of sexual transmission diseases (56, 99). Women’s diagnosis of cervical cancer is a turning point that requires the women to be linked to cancer clinics or start receiving cancer care. Women face several challenges (e.g., self-image and personality, financial challenges) in the health care system after being diagnosed with cervical cancer in Ethiopia. Similarly, a systematic review in low- and middle-income countries identified that cervical cancer results in financial insecurity among women and children (100). The impact of cervical cancer on children and families, on employment, education, and household income, needs further study in Ethiopia. Many women are unsatisfied with services, have an impaired quality of life, and face financial crises; they interrupt treatment and unattended health care. As a result, women with confirmed cervical cancer had a high chance of mortality or a low chance of survival in Ethiopia (72).

As to future research implications, there are several crucial topics that were not examined in the previous research. For example, the extent and effectiveness of community engagement and multisectoral collaboration towards cervical cancer service uptake and outcome improvement. The available evidence did not also provide insight about the acceptability and applicability of interventions which are beyond HPV and screening. There are a few interventional studies on knowledge and service uptake improvement that should be evaluated further to transform the response to cervical cancer towards its elimination. Almost all studies used a quantitative approach, and the participants of these studies were women. It is also crucial getting data from men, key informants, religious leaders, traditional healers, and policy document review.

### Strength and Limitation

This scoping review provides a comprehensive assessment of the literature on cervical cancer prevention and outcomes. As scoping reviews do not typically require quality appraisal of article, the authors did not evaluate the quality status of the included studies.

## Conclusions And Recommendation

Overall, there is inadequate knowledge, perception, health seeking behaviour, screening and treatment services. This implies that the country is lagging behind the targets towards eliminating cervical cancer. It is therefore crucial that the country identifies the key implementation issues, challenges and strategies to scale up both primary and secondary prevention services so that cervical cancer will not anymore be a public health problem. This requires a whole-of-society and a whole-of-government response based on the primary health care approach.

## Figures and Tables

**Figure 1:**
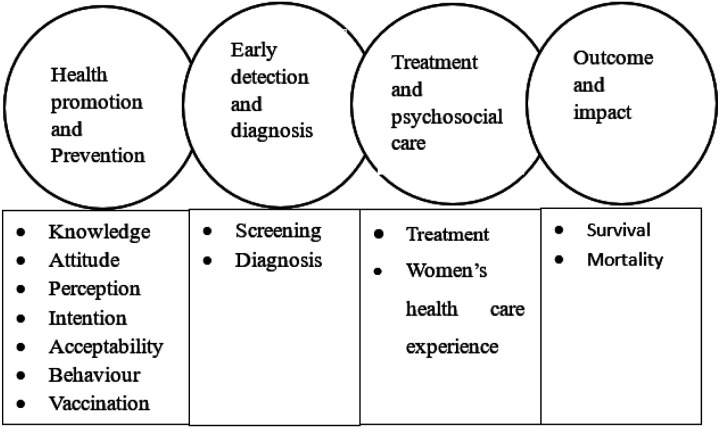
cervical cancer care/control continuum model used to report review findings

## Data Availability

The data set is available within this manuscript.

## Abbreviations

CHWs: Community Health Workers
PHC: Primary Health Care
UK: United Kingdom
UHC: Universal Health Coverage
UNs: United Nations
USA: United States of America
WHO: World Health Organisation

## References

[1] Sung H, Ferlay J, Siegel RL, Laversanne M, Soerjomataram I, Jemal A, Global cancer statistics 2020: GLOBOCAN estimates of incidence and mortality worldwide for 36 cancers in 185 countries. CA: a cancer journal for clinicians. 2021;71(3):209–49. 33538338 10.3322/caac.21660

[2] Ginsburg O, Bray F, Coleman MP, Vanderpuye V, Eniu A, Kotha SR, The global burden of women’s cancers: a grand challenge in global health. The Lancet. 2017;389(10071):847–60. 10.1016/S0140-6736(16)31392-7 PMC6191029 27814965

[3] Arbyn M, Weiderpass E, Bruni L, de Sanjosé S, Saraiya M, Ferlay J, Estimates of incidence and mortality of cervical cancer in 2018: a worldwide analysis. The Lancet Global Health. 2020;8(2):e191–e203. 31812369 10.1016/S2214-109X(19)30482-6 PMC7025157

[4] Bruni L, Albero G, Serrano B, Mena M, Collado JJ, Gómez D, Muñoz J, Bosch FX, de Sanjosé S. ICO/IARC Information Centre on HPV and Cancer (HPV Information Centre). Human Papillomavirus and Related Diseases in Ethiopia. Summary Report 10 March 2023.

[5] Zhang X, Zeng Q, Cai W, Ruan W. Trends of cervical cancer at global, regional, and national level: data from the Global Burden of Disease study 2019. BMC public health. 2021;21(1):1–10. 33975583 10.1186/s12889-021-10907-5 PMC8114503

[6] Kocarnik JM, Compton K, Dean FE, Fu W, Gaw BL, Harvey JD, Cancer incidence, mortality, years of life lost, years lived with disability, and disability-adjusted life years for 29 cancer groups from 2010 to 2019: a systematic analysis for the global burden of disease study 2019. JAMA oncology. 2022;8(3):420–44. 34967848 10.1001/jamaoncol.2021.6987 PMC8719276

[7] Abbas KM, van Zandvoort K, Brisson M, Jit M. Effects of updated demography, disability weights, and cervical cancer burden on estimates of human papillomavirus vaccination impact at the global, regional, and national levels: a PRIME modelling study. The Lancet Global Health. 2020;8(4):e536–e44. 32105613 10.1016/S2214-109X(20)30022-X PMC7083230

[8] World Health Organization. Global strategy to accelerate the elimination of cervical cancer as a public health problem 2020 [Available from: https://www.who.int/publications/i/item/9789240014107.

[9] The Economist Intelligence Unit. Global action on financing cervical cancer elimination Funding secondary prevention services in low resource settings 2020 [Available from: https://www.eiu.com/default.aspx.

[10] Union for International Cancer Control Cervical Cancer: a unique opportunity to work towards equity [Available from: https://www.uicc.org/what-we-do/driving-global-impact/targeted-commitments/cervical-cancer-elimination-strategy?gclid=CjwKCAjwoIqhBhAGEiwArXT7KxW6k5wQhHRpuApNn9os7N9GIyzf8aGvvZcr6IVdr-ccOihGV0PTGxoC_AUQAvD_BwE#49619.

[11] Lakneh EA, Mersha EA, Asresie MB, Belay HG. Knowledge, attitude, and uptake of human papilloma virus vaccine and associated factors among female preparatory school students in Bahir Dar City, Amhara Region, Ethiopia. Plos one. 2022;17(11):e0276465. 36409675 10.1371/journal.pone.0276465 PMC9678319

[12] Larebo YM, Elilo LT, Abame DE, Akiso DE, Bawore SG, Anshebo AA, Awareness, Acceptance, and Associated Factors of Human Papillomavirus Vaccine among Parents of Daughters in Hadiya Zone, Southern Ethiopia: A Cross-Sectional Study. Vaccines. 2022;10(12):1988. 36560398 10.3390/vaccines10121988 PMC9785952

[13] (DCCPS) DoCCaPS. Cancer Control Continuum: National Cancer Institute; 2020 [Available from: https://cancercontrol.cancer.gov/about-dccps/about-cc/cancer-control-continuum.

[14] Bekele HT, Nuri A, Abera L. Knowledge, Attitude, and Practice Toward Cervical Cancer Screening and Associated Factors Among College and University Female Students in Dire Dawa City, Eastern Ethiopia. Cancer Inform. 2022;21:11769351221084808. 10.1177/11769351221084808 PMC8998372 35418740

[15] Mruts KB, Gebremariam TB. Knowledge and Perception Towards Cervical Cancer among Female Debre Berhan University Students. Asian Pac J Cancer Prev. 2018;19(7):1771–7. 30049186 10.22034/APJCP.2018.19.7.1771 PMC6165663

[16] Abebaw E, Tesfa M, Gezimu W, Bekele F, Duguma A. Female healthcare providers’ knowledge, attitude, and practice towards cervical cancer screening and associated factors in public hospitals of Northwest Ethiopia. SAGE Open Med. 2022;10:20503121221095931. 10.1177/20503121221095931 PMC9118899 35600715

[17] Ararsa T, Tadele N, Ayalew Y, Gela D. Knowledge towards cervical cancer screening and associated factors among urban health extension workers at Addis Ababa, Ethiopia: facility based cross-sectional survey. BMC Cancer. 2021;21(1):224. 33663412 10.1186/s12885-021-07952-z PMC7934429

[18] Erku DA, Netere AK, Mersha AG, Abebe SA, Mekuria AB, Belachew SA. Comprehensive knowledge and uptake of cervical cancer screening is low among women living with HIV/AIDS in Northwest Ethiopia. Gynecol Oncol Res Pract. 2017;4:20. 29276611 10.1186/s40661-017-0057-6 PMC5738137

[19] Dulla D, Daka D, Wakgari N. Knowledge about cervical cancer screening and its practice among female health care workers in Southern Ethiopia: A cross-sectional study. International Journal of Women’s Health. 2017;9:365–72. 10.2147/IJWH.S132202 PMC5446960 28579837

[20] Mengesha A, Messele A, Beletew B. Knowledge and attitude towards cervical cancer among reproductive age group women in Gondar town, North West Ethiopia. BMC Public Health. 2020;20(1):209. 32046681 10.1186/s12889-020-8229-4 PMC7014652

[21] Tsegay A, Araya T, Amare K, G/tsadik F. Knowledge, attitude, and practice on cervical cancer screening and associated factors among women aged 15–49 years in Adigrat Town, Northern Ethiopia, 2019: a Community-Based Cross-Sectional Study. International Journal of Women’s Health. 2021:1283–98. 10.2147/IJWH.S261204 PMC7801815 33447089

[22] Tilahun T, Tulu T, Dechasa W. Knowledge, attitude and practice of cervical cancer screening and associated factors amongst female students at Wollega University, western Ethiopia. BMC Res Notes. 2019;12(1):518. 31426860 10.1186/s13104-019-4564-x PMC6701027

[23] Tadesse A, Tafa Segni M, Demissie HF. Knowledge, Attitude, and Practice (KAP) toward Cervical Cancer Screening among Adama Science and Technology University Female Students, Ethiopia. International Journal of Breast Cancer. 2022;2022. 10.1155/2022/2490327 PMC8776479 35070454

[24] Tafere Y, Jemere T, Desalegn T, Melak A. Women’s knowledge and attitude towards cervical cancer preventive measures and associated factors In South Gondar Zone, Amhara Region, North Central Ethiopia: a cross-sectional study. Arch Public Health. 2021;79(1):136. 34301336 10.1186/s13690-021-00659-4 PMC8299606

[25] Gebisa T, Bala ET, Deriba BS. Knowledge, Attitude, and Practice Toward Cervical Cancer Screening Among Women Attending Health Facilities in Central Ethiopia. Cancer Control. 2022;29:10732748221076680. 10.1177/10732748221076680 PMC8943579 35315704

[26] Aweke YH, Ayanto SY, Ersado TL. Knowledge, attitude and practice for cervical cancer prevention and control among women of childbearing age in Hossana Town, Hadiya zone, Southern Ethiopia: Community-based cross-sectional study. PLoS ONE. 2017;12(7). 10.1371/journal.pone.0181415 PMC5526548 28742851

[27] Demissie BW, Azeze GA, Asseffa NA, Lake EA, Besha BB, Gelaw KA, Communities’ perceptions towards cervical cancer and its screening in Wolaita zone, southern Ethiopia: A qualitative study. PLoS One. 2022;17(1):e0262142. 34995307 10.1371/journal.pone.0262142 PMC8740975

[28] Birhanu Z, Abdissa A, Belachew T, Deribew A, Segni H, Tsu V, Health seeking behavior for cervical cancer in Ethiopia: A qualitative study. International Journal for Equity in Health. 2012;11(1). 10.1186/1475-9276-11-83 PMC3544623 23273140

[29] Mesafint Z, Berhane Y, Desalegn D. Health Seeking Behavior of Patients Diagnosed with Cervical Cancer in Addis Ababa, Ethiopia. Ethiopian journal of health sciences. 2018;28(2):111–6. 29983508 10.4314/ejhs.v28i2.2 PMC6016345

[30] Habtu Y, Yohannes S, Laelago T. Health seeking behavior and its determinants for cervical cancer among women of childbearing age in Hossana Town, Hadiya zone, Southern Ethiopia: community based cross sectional study. BMC Cancer. 2018;18(1):298. 29548313 10.1186/s12885-018-4203-2 PMC5857120

[31] Mihretie GN, Liyeh TM, Ayele AD, Belay HG, Yimer TS, Miskr AD. Knowledge and willingness of parents towards child girl HPV vaccination in Debre Tabor Town, Ethiopia: a community-based cross-sectional study. Reprod Health. 2022;19(1):136. 35689288 10.1186/s12978-022-01444-4 PMC9188100

[32] Dereje N, Ashenafi A, Abera A, Melaku E, Yirgashewa K, Yitna M, Knowledge and acceptance of HPV vaccination and its associated factors among parents of daughters in Addis Ababa, Ethiopia: a community-based cross-sectional study. Infect Agent Cancer. 2021;16(1):58. 34479576 10.1186/s13027-021-00399-8 PMC8418033

[33] Beyen MW, Bulto GA, Chaka EE, Debelo BT, Roga EY, Wakgari N, Human papillomavirus vaccination uptake and its associated factors among adolescent school girls in Ambo town, Oromia region, Ethiopia, 2020. PLoS One. 2022;17(7):e0271237. 35830389 10.1371/journal.pone.0271237 PMC9278730

[34] Kassa HN, Bilchut AH, Mekuria AD, Lewetie EM. Practice and Associated Factors of Human Papillomavirus Vaccination Among Primary School Students in Minjar-Shenkora District, North Shoa Zone, Amhara Regional State, Ethiopia, 2020. Cancer Manag Res. 2021;13:6999–7008. 34522142 10.2147/CMAR.S324078 PMC8434827

[35] Aragaw GM, Anteneh TA, Abiy SA, Bewota MA, Aynalem GL. Parents’ willingness to vaccinate their daughters with human papillomavirus vaccine and associated factors in Debretabor town, Northwest Ethiopia: A community-based cross-sectional study. Human Vaccines & Immunotherapeutics. 2023:2176082. 36794293 10.1080/21645515.2023.2176082 PMC10026865

[36] Ukumo EY, Weldehawariat FG, Dessalegn SA, Minamo DM, Weldehawaryat HN. Acceptance of Human Papillomavirus Vaccination and Associated Factors among Girls in Arba Minch Town, Southern Ethiopia, 2020. Infect Dis Obstet Gynecol. 2022;2022:7303801. 36531338 10.1155/2022/7303801 PMC9750771

[37] Alene T, Atnafu A, Mekonnen ZA, Minyihun A. Acceptance of human papillomavirus vaccination and associated factors among parents of daughters in gondar town, northwest Ethiopia. Cancer Management and Research. 2020;12:8519–26. 32982444 10.2147/CMAR.S275038 PMC7502398

[38] Biyazin T, Yetwale A, Fenta B. Willingness to accept human papillomavirus vaccination in Jimma town, Ethiopia. Hum Vaccin Immunother. 2022;18(6):2125701. 36161874 10.1080/21645515.2022.2125701 PMC9746436

[39] Desta M, Getaneh T, Yeserah B, Worku Y, Eshete T, Birhanu MY, Cervical cancer screening utilization and predictors among eligible women in Ethiopia: A systematic review and meta-analysis. PLoS One. 2021;16(11):e0259339. 34735507 10.1371/journal.pone.0259339 PMC8568159

[40] Dessalegn Mekonnen B. Cervical Cancer Screening Uptake and Associated Factors among HIV-Positive Women in Ethiopia: A Systematic Review and Meta-Analysis. Adv Prev Med. 2020;2020:7071925. 32879739 10.1155/2020/7071925 PMC7448202

[41] Argaw M, Embiale A, Amare B. Knowledge, and practice of cervical cancer prevention and associated factors among commercial sex workers in Shashemene Town, West Arsi, Oromia Region, Ethiopia. BMC Womens Health. 2022;22(1):233. 35710370 10.1186/s12905-022-01819-6 PMC9205103

[42] Wasiyhun TS, Beyene MG, Deghebo AD, Getachew T, Zeleke GT, Tesema TT, Availability and readiness of cervical cancer screening service at health facilities in Ethiopia. Ethiopian Journal of public health and nutrition. 2021;4(2):141= 5-= 5.

[43] Jede F, Brandt T, Gedefaw M, Wubneh SB, Abebe T, Teka B, Home-based HPV self-sampling assisted by a cloud-based electronic data system: Lessons learnt from a pilot community cervical cancer screening campaign in rural Ethiopia. Papillomavirus Res. 2020;9:100198. 32416283 10.1016/j.pvr.2020.100198 PMC7240728

[44] de Fouw M, Kaba M, Hailu M, Bereket FZ, Beltman JJ. Local community networks in the fight against cervical cancer: the role of coffee ceremonies in the uptake of screening in Ethiopia. Trop Doct. 2019;49(4):298–300. 31333059 10.1177/0049475519864763 PMC6961085

[45] Abu SH, Woldehanna BT, Nida ET, Tilahun AW, Gebremariam MY, Sisay MM. The role of health education on cervical cancer screening uptake at selected health centers in Addis Ababa. PLoS One. 2020;15(10):e0239580. 33027267 10.1371/journal.pone.0239580 PMC7540882

[46] Shiferaw N, Salvador-Davila G, Kassahun K, Brooks MI, Weldegebreal T, Tilahun Y, The Single-Visit Approach as a Cervical Cancer Prevention Strategy Among Women With HIV in Ethiopia: Successes and Lessons Learned. Global health, science and practice. 2016;4(1):87–98. 27016546 10.9745/GHSP-D-15-00325 PMC4807751

[47] Assefa T, Arefaynie M, Mebratu W, Mohammed A, Addisu E, Kebede N. Determinants of cervical cancer screening utilization among women attending health facilities of Dessie town, Northeast Ethiopia. BMC Cancer. 2022;22(1):1330. 36539726 10.1186/s12885-022-10447-0 PMC9764547

[48] Ayenew AA, Zewdu BF, Nigussie AA. Uptake of cervical cancer screening service and associated factors among age-eligible women in Ethiopia: systematic review and meta-analysis. Infect Agent Cancer. 2020;15(1):67. 33292388 10.1186/s13027-020-00334-3 PMC7666476

[49] Midaksa M, Destaw A, Addissie A, Kantelhardt EJ, Gizaw M. Women’s sexual autonomy as a determinant of cervical cancer screening uptake in Addis Ababa, Ethiopia: a case–control study. BMC Women’s Health. 2022;22(1):236. 35715797 10.1186/s12905-022-01829-4 PMC9206386

[50] Lott BE, Halkiyo A, Kassa DW, Kebede T, Dedefo A, Ehiri J, Health workers’ perspectives on barriers and facilitators to implementing a new national cervical cancer screening program in Ethiopia. BMC Women’s Health. 2021;21(1). 10.1186/s12905-021-01331-3 PMC8090515 33941159

[51] Megersa BS, Bussmann H, Bärnighausen T, Muche AA, Alemu K, Deckert A. Community cervical cancer screening: Barriers to successful home-based HPV self-sampling in Dabat district, North Gondar, Ethiopia. A qualitative study. PLoS One. 2020;15(12):e0243036. 33306681 10.1371/journal.pone.0243036 PMC7732077

[52] Temesgen MM, Alemu T, Shiferaw B, Legesse S, Zeru T, Haile M, Prevalence of oncogenic human papillomavirus (HPV 16/18) infection, cervical lesions and its associated factors among women aged 21–49 years in Amhara region, Northern Ethiopia. Plos one. 2021;16(3):e0248949. 33760866 10.1371/journal.pone.0248949 PMC7990306

[53] Fentie AM, Tadesse TB, Gebretekle GB. Factors affecting cervical cancer screening uptake, visual inspection with acetic acid positivity and its predictors among women attending cervical cancer screening service in Addis Ababa, Ethiopia. BMC Womens Health. 2020;20(1):147. 32677933 10.1186/s12905-020-01008-3 PMC7366887

[54] Mekuria M, Edosa K, Endashaw M, Bala ET, Chaka EE, Deriba BS, Prevalence of Cervical Cancer and Associated Factors Among Women Attended Cervical Cancer Screening Center at Gahandi Memorial Hospital, Ethiopia. Cancer Inform. 2021;20:11769351211068431. 10.1177/11769351211068431 PMC8725021 34992337

[55] Getinet M, Taye M, Ayinalem A, Gitie M. Precancerous Lesions of the Cervix and Associated Factors among Women of East Gojjam, Northwest Ethiopia, 2020. Cancer Manag Res. 2021;13:9401–10. 35002317 10.2147/CMAR.S338177 PMC8721437

[56] Belayneh T, Mitiku H, Weldegebreal F. Precancerous cervical lesion and associated factors among HIV-infected women on ART in Amhara Regional State, Ethiopia: A hospital-based cross-sectional study. Int J Health Sci (Qassim). 2019;13(3):4–9. PMC6512148 31123433

[57] Pelzer A, Duncan ME, Tibaux G, Mehari L. A study of cervical cancer in Ethiopian women. Cytopathology. 1992;3(3):139–48. 1511118 10.1111/j.1365-2303.1992.tb00039.x

[58] Zelalem W, Weldegebreal F, Ayele BH, Deressa A, Debella A, Eyeberu A, Precancerous Cervical Lesion Among Adult Women With Human Immune Deficiency Virus on Anti Retroviral Therapy At Saint Peter Specialized Hospital, Ethiopia: A Hospital-Based Cross-Sectional Study. Front Oncol. 2022;12:910915. 35957869 10.3389/fonc.2022.910915 PMC9361014

[59] Begoihn M, Mathewos A, Aynalem A, Wondemagegnehu T, Moelle U, Gizaw M, Cervical cancer in Ethiopia–predictors of advanced stage and prolonged time to diagnosis. Infectious agents and cancer. 2019;14(1):1–7. 31737087 10.1186/s13027-019-0255-4 PMC6849163

[60] Dereje N, Addissie A, Worku A, Gebremariam A, Kantelhardt EJ, Assefa M, Association between waiting time for radiotherapy initiation and disease progression among women with cervical cancer in Addis Ababa, Ethiopia. International Journal of Cancer. 2021;149(6):1284–9. 33997978 10.1002/ijc.33689

[61] Zeleke S, Anley M, Kefale D, Wassihun B. Factors Associated with Delayed Diagnosis of Cervical Cancer in Tikur Anbesa Specialized Hospital, Ethiopia, 2019: Cross-Sectional Study. Cancer Manag Res. 2021;13:579–85. 33519237 10.2147/CMAR.S285621 PMC7837583

[62] Atnafu T, Daka DW, Debela TF, Ergiba MS. Women’s Satisfaction with Cervical Cancer Screening Services and Associated Factors in Maternal Health Clinics of Jimma Town Public Health Facilities, Southwest Ethiopia. Cancer Manag Res. 2021;13:7685–96. 34675665 10.2147/CMAR.S327369 PMC8504707

[63] Dirar A, Mekonnen W, Berhanu Z. The Experiences of Cervical Cancer Patients During Follow-Up Care in Ethiopia: A Qualitative Study. Cancer Manag Res. 2022;14:2507–18. 36035503 10.2147/CMAR.S373379 PMC9416456

[64] Kebebew T, Mosalo A, Mavhandu-Mudzusi AH. Caregivers’ satisfaction with cervical cancer care in Ethiopia. Support Care Cancer. 2022;30(9):7597–603. 35674793 10.1007/s00520-022-07201-4

[65] Endale H, Mulugeta T, Habte T. The Socioeconomic Impact of Cervical Cancer on Patients in Ethiopia: Evidence from Tikur Anbessa Specialized Hospital. Cancer Manag Res. 2022;14:1615–25. 35535268 10.2147/CMAR.S352389 PMC9078746

[66] Araya LT, Fenta TG, Sander B, Gebremariam GT, Gebretekle GB. Health-related quality of life and associated factors among cervical cancer patients at Tikur Anbessa specialized hospital, Addis Ababa, Ethiopia. Health and Quality of Life Outcomes. 2020;18(1). 10.1186/s12955-020-01319-x PMC7076924 32178681

[67] Hailu A, Mariam DH. Patient side cost and its predictors for cervical cancer in Ethiopia: A cross sectional hospital based study. BMC Cancer. 2013;13. 10.1186/1471-2407-13-69 PMC3576296 23391288

[68] Deressa BT, Assefa M, Tafesse E, Kantelhardt EJ, Soldatovic I, Cihoric N, Contemporary treatment patterns and survival of cervical cancer patients in Ethiopia. BMC Cancer. 2021;21(1). 10.1186/s12885-021-08817-1 PMC8515694 34645407

[69] Wassie M, Argaw Z, Tsige Y, Abebe M, Kisa S. Survival status and associated factors of death among cervical cancer patients attending at Tikur Anbesa Specialized Hospital, Addis Ababa, Ethiopia: a retrospective cohort study. BMC Cancer. 2019;19(1):1221. 31842805 10.1186/s12885-019-6447-x PMC6916089

[70] Wassie M, Fentie B. Prevalence of late-stage presentation and associated factors of cervical cancer patients in Tikur Anbesa Specialized Hospital, Ethiopia: institutional based cross-sectional study. Infectious Agents and Cancer. 2021;16(1). 10.1186/s13027-021-00371-6 PMC8111725 33975620

[71] Seifu B, Fikru C, Yilma D, Tessema F. Predictors of time to death among cervical cancer patients at Tikur Anbesa specialized hospital from 2014 to 2019: A survival analysis. PLoS One. 2022;17(2):e0264369. 35202442 10.1371/journal.pone.0264369 PMC8870501

[72] Gashu C, Tasfa B, Alemu C, Kassa Y. Assessing survival time of outpatients with cervical cancer: at the university of Gondar referral hospital using the Bayesian approach. BMC Womens Health. 2023;23(1):59. 36765315 10.1186/s12905-023-02202-9 PMC9921662

[73] Aguade AE, Gashu C, Jegnaw T. The trend of change in cervical tumor size and time to death of hospitalized patients in northwestern Ethiopia during 2018–2022: Retrospective study design. Health Sci Rep. 2023;6(2):e1121. 36814966 10.1002/hsr2.1121 PMC9939582

[74] Mebratie AE, Moges NA, Meselu BT, Melesse MF. Time to death from cervical cancer and predictors among cervical cancer patients in Felege Hiwot Comprehensive Specialized Hospital, North West Ethiopia: Facility-based retrospective follow-up study. PLoS One. 2022;17(6):e0269576. 35749539 10.1371/journal.pone.0269576 PMC9232151

[75] Moelle U, Mathewos A, Aynalem A, Wondemagegnehu T, Yonas B, Begoihn M, Cervical Cancer in Ethiopia: The Effect of Adherence to Radiotherapy on Survival. Oncologist. 2018;23(9):1024–32. 29567823 10.1634/theoncologist.2017-0271 PMC6192604

[76] Gizaw M, Addissie A, Getachew S, Ayele W, Mitiku I, Moelle U, Cervical cancer patients presentation and survival in the only oncology referral hospital, Ethiopia: A retrospective cohort study. Infectious Agents and Cancer. 2017;12(1). 10.1186/s13027-017-0171-4 PMC5708091 29213299

[77] Getaneh A, Tegene B, Belachew T. Knowledge, attitude and practices on cervical cancer screening among undergraduate female students in University of Gondar, Northwest Ethiopia: an institution based cross sectional study. BMC Public Health. 2021;21(1):775. 33888094 10.1186/s12889-021-10853-2 PMC8063279

[78] Wakwoya EB, Gemechu KS, Dasa TT. Knowledge of Cervical Cancer and Associated Factors Among Women Attending Public Health Facilities in Eastern Ethiopia. Cancer Manag Res. 2020;12:10103–11. 33116866 10.2147/CMAR.S262314 PMC7569063

[79] Chali K, Oljira D, Sileshi T, Mekonnen T. Knowledge on cervical cancer, attitude toward its screening, and associated factors among reproductive age women in Metu Town, Ilu Aba Bor, South West Ethiopia, 2018: community-based cross-sectional study. Cancer Rep (Hoboken). 2021;4(5):e1382. 33934571 10.1002/cnr2.1382 PMC8552000

[80] Tafere Y, Jemere T, Desalegn T, Melak A. Women’s knowledge and attitude towards cervical cancer preventive measures and associated factors In South Gondar Zone, Amhara Region, North Central Ethiopia: a cross-sectional study. Archives of Public Health. 2021;79(1):1–7. 34301336 10.1186/s13690-021-00659-4 PMC8299606

[81] Tekle T, Wolka E, Nega B, Kumma WP, Koyira MM. Knowledge, Attitude and Practice Towards Cervical Cancer Screening Among Women and Associated Factors in Hospitals of Wolaita Zone, Southern Ethiopia. Cancer Manag Res. 2020;12:993–1005. 32104081 10.2147/CMAR.S240364 PMC7023884

[82] Bogale AL, Teklehaymanot T, Haidar Ali J, Kassie GM. Knowledge, attitude and practice of cervical cancer screening among women infected with HIV in Africa: Systematic review and meta-analysis. PLoS One. 2021;16(4):e0249960. 33831128 10.1371/journal.pone.0249960 PMC8031808

[83] Belete N, Tsige Y, Mellie H. Willingness and acceptability of cervical cancer screening among women living with HIV/AIDS in Addis Ababa, Ethiopia: a cross sectional study. Gynecol Oncol Res Pract. 2015;2:6. 27231566 10.1186/s40661-015-0012-3 PMC4881166

[84] Eshete M, Abdulwuhab Atta M, Yeshita HY. Cervical Cancer Screening Acceptance among Women in Dabat District, Northwest Ethiopia, 2017: An Institution-Based Cross-Sectional Study. Obstet Gynecol Int. 2020;2020:2805936. 32089698 10.1155/2020/2805936 PMC7029298

[85] Ampofo AG, Boyes AW, Khumalo PG, Mackenzie L. Improving knowledge, attitudes, and uptake of cervical cancer prevention among female students: A systematic review and meta-analysis of school-based health education. Gynecologic Oncology. 2022. 10.1016/j.ygyno.2021.12.021 34998599

[86] Ortenzi F, Marten R, Valentine NB, Kwamie A, Rasanathan K. Whole of government and whole of society approaches: call for further research to improve population health and health equity. BMJ Specialist Journals; 2022. p. e009972. 10.1136/bmjgh-2022-009972 PMC9344990 35906017

[87] Spayne J, Hesketh T. Estimate of global human papillomavirus vaccination coverage: Analysis of country-level indicators. BMJ open. 2021;11(9):e052016. 10.1136/bmjopen-2021-052016 PMC8413939 34475188

[88] Dorji T, Nopsopon T, Tamang ST, Pongpirul K. Human papillomavirus vaccination uptake in low-and middle-income countries: a meta-analysis. EClinicalMedicine. 2021;34:100836. 33997733 10.1016/j.eclinm.2021.100836 PMC8102703

[89] Mavundza EJ, Iwu-Jaja CJ, Wiyeh AB, Gausi B, Abdullahi LH, Halle-Ekane G, A systematic review of interventions to improve HPV vaccination coverage. Vaccines. 2021;9(7):687. 34201421 10.3390/vaccines9070687 PMC8310215

[90] Belay Y, Dheresa M, Sema A, Desalew A, Assefa N. Cervical Cancer Screening Utilization and Associated Factors Among Women Aged 30 to 49 Years in Dire Dawa, Eastern Ethiopia. Cancer Control. 2020;27(1). 10.1177/1073274820958701 PMC7791449 33034204

[91] Kassie AM, Abate BB, Kassaw MW, Aragie TG, Geleta BA, Shiferaw WS. Impact of knowledge and attitude on the utilization rate of cervical cancer screening tests among Ethiopian women: A systematic review and meta-analysis. PLoS One. 2020;15(12):e0239927. 33290426 10.1371/journal.pone.0239927 PMC7723289

[92] Bruni L, Serrano B, Roura E, Alemany L, Cowan M, Herrero R, Cervical cancer screening programmes and age-specific coverage estimates for 202 countries and territories worldwide: a review and synthetic analysis. The Lancet Global Health. 2022;10(8):e1115–e27. 35839811 10.1016/S2214-109X(22)00241-8 PMC9296658

[93] Amado G, Weldegebreal F, Birhanu S, Dessie Y. Cervical cancer screening practices and its associated factors among females of reproductive age in Durame town, Southern Ethiopia. PLoS One. 2022;17(12):e0279870. 36584208 10.1371/journal.pone.0279870 PMC9803181

[94] Tefera F, Mitiku I. Uptake of Cervical Cancer Screening and Associated Factors Among 15–49-Year-Old Women in Dessie Town, Northeast Ethiopia. J Cancer Educ. 2017;32(4):901–7. 27075197 10.1007/s13187-016-1021-6

[95] Mezei AK, Armstrong HL, Pedersen HN, Campos NG, Mitchell SM, Sekikubo M, Cost-effectiveness of cervical cancer screening methods in low-and middle-income countries: A systematic review. International journal of cancer. 2017;141(3):437–46. 28297074 10.1002/ijc.30695

[96] Robbers GML, Bennett LR, Spagnoletti BRM, Wilopo SA. Facilitators and barriers for the delivery and uptake of cervical cancer screening in Indonesia: a scoping review. Global Health Action. 2021;14(1):1979280. 34586032 10.1080/16549716.2021.1979280 PMC8491705

[97] Gebresilasie SF, Zegeye A. Accuracy of VIA for the diagnosis of cervical cancer and associated factors among women attending cervical cancer screening at Hawassa university comprehensive specialized hospital, southern Ethiopia: Institutional based cross sectional study. Ann Med Surg (Lond). 2022;84:104873. 36536735 10.1016/j.amsu.2022.104873 PMC9758352

[98] Hull R, Mbele M, Makhafola T, Hicks C, Wang SM, Reis RM, Cervical cancer in low and middle-income countries. Oncology letters. 2020;20(3):2058–74. 32782524 10.3892/ol.2020.11754 PMC7400218

[99] Zena D, Elfu B, Mulatu K. Prevalence and Associated Factors of Precancerous Cervical Lesions among Women in Ethiopia: A Systematic Review and Meta-Analysis. Ethiop J Health Sci. 2021;31(1):189–200. 34158766 10.4314/ejhs.v31i1.21 PMC8188114

[100] Dau H, Trawin J, Nakisige C, Payne BA, Vidler M, Singer J, The social and economic impacts of cervical cancer on women and children in low-and middle-income countries: A systematic review. International Journal of Gynecology & Obstetrics. 2023;160(3):751–61. 35962711 10.1002/ijgo.14395

